# The HLA-B*57:01 Haplotype, Genetic Key Linking Innate Immunity and Longevity

**DOI:** 10.14336/AD.2025.1109

**Published:** 2026-10-14

**Authors:** Myriam Rahmouni, Cristina Giuliani, Daniela Colucci, Nicolás Mendoza-Mejía, Samuel Hiet, Marco Lamanda, Jean-Louis Spadoni, Sigrid Le Clerc, Taoufik Labib, Matilde Manetti, Maxime Tison, Raissa Medina-Santos, Paolo Garagnani, Alexandre How-Kit, Armand Bensussan, Marianne Nygaard, Kaare Christensen, Almut Nebel, Jean-François Deleuze, Jean-François Zagury

**Affiliations:** ^1^Laboratoire Génomique, Bioinformatique, et Chimie Moléculaire, EA7528, Conservatoire National des Arts et Métiers, Paris, France.; ^2^Laboratory of Molecular Anthropology, Department of Biological, Geological and Environmental Sciences (BiGeA), University of Bologna, Bologna, Italy.; ^3^Institute of Clinical Molecular Biology, Kiel University, Kiel, Germany.; ^4^IRCCS Azienda Ospedaliero-Universitaria di Bologna, Bologna, Italy.; ^5^Dipartimento di Scienze Mediche e Chirurgiche, Università di Bologna, Bologna, Italy.; ^6^Laboratory for Genomics, Foundation Jean Dausset - CEPH, Paris, France.; ^7^Unité INSERM U976, Hôpital Saint-Louis, Paris, France.; ^8^Epidemiology, Biostatistics and Biodemography, Department of Public Health, University of Southern Denmark, Odense, Denmark.; ^9^Department of Clinical Genetics, Odense University Hospital, Odense, Denmark.; ^10^Department of Clinical Biochemistry, Odense University Hospital, Odense, Denmark.

**Keywords:** HLA Antigens, MICA, MICB, HLA-B*57, centenarian, longevity

## Abstract

In our recent research on AIDS Elite controllers, we demonstrated that the HLA-B*57:01 allele is part of an extensive MHC haplotype associated with impaired MICA/MICB function. Considering the pivotal role of natural killer (NK) cells and innate immunity in fighting cancer and infectious diseases, we investigated whether this allele could also influence human longevity. To this end, we performed a multinational European study including 2,597 centenarians and 9,973 controls. We found that HLA-B*57:01 was consistently less frequent in the European centenarian cohorts compared with the corresponding controls (OR ≈ 0.68, p < 0.0005). These findings provide the first in vivo genetic evidence that innate immunity and NK cell function are key components of human longevity.

## INTRODUCTION

HLA-B*57:01 is well-known for its association with the control of HIV-1 viral load and non-progression in AIDS [1]. Several studies have confirmed the potency of this HLA allele in AIDS through its impact on HLA class I genes, either through their overexpression or through the protective presentation of HIV-1 peptides to the immune system [1]. In 2014, our group compared 50 HIV-1 elite controllers from the GRIV cohort with uninfected controls and pointed out that the strongest association signal, SNP rs2395029-G (tagSNP for the allele HLA-B*57:01), corresponded to a haplotype of the Major Histocompatibility Complex in high linkage disequilibrium (LD) (r^2^=0.88 in Europeans) with the deletion rs199503730, which tags the MICA*017 allele, and suggested that this allele might be not functional [2]. Shortly thereafter, MICA*017 was indeed shown experimentally to be dysfunctional by disrupting splicing, leading to the absence of the full-length MICA transcript and the production of truncated isoforms [3]. More recently, by analyzing the MHC haploblock associations in a much larger cohort of HIV-1 elite controllers (N=543) from the ICGH consortium [4], we have reconfirmed the association with the MICA*017 allele and showed additionally that the rs2395029-G/HLA-B*57:01 allele was also associated with a decrease in MICB expression [5]. The HLA-B*57:01 haplotype thus affects the recognition of MICA by its ligand NKG2D on NK cells and decreases the expression of MICB, leading to a partial dysfunction of the MICA/MICB induced-NK activating signaling through NKG2D [5]. Since it is known that HIV-1 escapes the first line of innate immunity mediated by NK cells through the shedding of soluble MICA/MICB by infected cells [6, 7], the MICA/MICB partial dysfunction could be a mechanism by which the HLA-B*57:01 haplotype favors the emergence of elite control during HIV-1 infection [5].

Importantly, the HLA-B*57:01 haplotype does not completely abolish the NK lymphocyte function since there is a second chromosome with possibly active forms of MICA/MICB and other functional activating NK receptors in the genome such as CD160 [8, 9]. Nevertheless, at the human population level, alterations in the MICA/MICB axis associated with the HLA-B*57:01 haplotype could exert epidemiological consequences. Given the well-established role of NK cells in combating cancer and infectious diseases [10], we hypothesized that this haplotype might influence human longevity. To test this, we evaluated whether the frequency of the rs2395029-G (tagSNP for the allele HLA- B*57:01) allele was reduced among European centenarians compared to controls of the same origin.

## MATERIALS AND METHODS

### Cohorts

This study analyzed four populations of European ancestry: French, Danish, Germans, and Italians.

#### French cohorts

The French case cohort was the CEPH Aging cohort (also called the CHRONOS cohort), a prospective study that includes the 1067 unrelated centenarians analyzed in our study. The CEPH Aging cohort comprises a total of 2,351 individuals (71.03% women) from 1,423 families, including 1,412 unrelated long-lived individuals (≥90 years) and 1,212 unrelated centenarians [11]. All participants provided written informed consent. The study design, recruitment strategy, clinical follow-up, data collection, and biobanking procedures are described in detail in the original publication [11]. Our analysis included 1,067 centenarians genotyped using the Illumina Human660W-Quad_v1_A array, which includes the SNP rs2395029

As French controls, we used two well-characterized cohorts: DESIR and SU.VI.MAX. The DESIR cohort (Data from an Epidemiological Study on the Insulin Resistance Syndrome) is a 9-year longitudinal study designed to investigate the development of insulin resistance and related metabolic disorders. It recruited 5212 individuals from the general French population, aged 30-64 years at inclusion (1994-1996) [12]. A sub-group of 693 controls with mean age: 34 ± 9 years and composed of 59% women was genotyped using the Illumina Infinium II HumanHap300 BeadChip (Illumina, San Diego, CA, USA), that contains the rs2395029 SNP [13].

The SU.VI.MAX cohort (Supplémentation en Vitamines et Minéraux Antioxydants) is a randomized, double-blind, placebo-controlled trial initiated in 1994 to evaluate the effects of daily antioxidant supplementation on the prevention of major chronic diseases [14]. Among the 12,735 participants enrolled, a sub-cohort of 1,823 French individuals (1,081 women aged 35-60 years and 742 men aged 45-60 years) was genotyped using the Illumina Infinium II HumanHap300 BeadChip (Illumina, San Diego, CA), that contains the rs2395029 SNP.

#### German Cohorts

The German longevity sample analyzed in this study comprised 692 unrelated centenarians (mean age 101.3 years), of which 80.3% were female. They were recruited from across Germany as described in a previous study [15]. The control group consisted of 6,332 unrelated younger Germans [16] (<83 years; mean age 57.2 years). Both cases and controls were genotyped using the Immunochip (Illumina, Inc., CA), and standard quality control procedures were rigorously applied [16]. SNP rs2395029 was present on the Immunochip and directly called.

#### Danish Cohorts

The Danish case group consisted of 505 unrelated centenarians (79% women) drawn from the 1905, 1910, 1911-12, and 1915 birth cohort studies [17, 18], the study of Danish Old Sibs (DOS), and the Longitudinal Study of Aging Danish Twins (LSADT) [19].

The control group consisted of 430 unrelated individuals younger than 80 years (45% women) selected from the Middle Age Danish Twins (MADT) Study [19]. Cases were genotyped using the Illumina Human OmniExpress Array (Illumina, San Diego, CA, USA), and controls using the Illumina Infinium PsychArray (Illumina, San Diego, CA, USA). Standard quality control procedures and genotype imputation were rigorously applied and have been described in detail [20]. The SNP rs2395029 was genotyped in controls and imputed in cases to the Type 1 Diabetes Genetics Consortium (T1DGC) reference panel [21] using SNP2HLA [22].

#### Italian Cohorts

The Italian cohort includes 333 unrelated centenarians (77 % women, mean age: 100.4 ± 1.4; 258 F, 75 M) from the 1910-1915 birth cohort and sampled from Northern, Central and Southern Italy at three different Italian recruitment centres in Northern Italy (N = 66), Central Italy (N= 176) and Southern Italy (N =91) [23].

Control group includes 773 geographically matched unrelated healthy individuals (46% women, < 82 years, mean age: 56.2 ± 11.5 years) who have been demonstrated to be highly representative of the overall Italian population.

Genotyping was performed using the Illumina CoreExomeChip v1.1 array (Illumina® Inc., San Diego, CA, USA) which includes the core set of ~280,000 genome-wide SNPs usually implemented in Illumina genotyping arrays, ~250,000 exomic markers and ~20,000 disease-associated variants.

Standard quality control procedures were applied as described in detail in the reference study [23]. The SNP rs2395029 was present in the array used and directly genotyped in both cases and controls.

### Statistical Analysis

For each of the four populations analyzed in this study, a population stratification analysis was performed. To assess genetic ancestry, we merged our datasets with the 1000 Genomes Project reference panel and conducted a principal component analysis (PCA). In all populations, individuals (combined cases and controls) clustered tightly with European reference populations, confirming their European genetic background. PCA was also used to verify that cases and controls were geographically matched within each population.

We used rs2395029 to assess HLA-B*57:01 in cases and controls, as it is known to be a highly reliable proxy for HLA-B*57:01 in European populations [24]. In addition, we imputed HLA-B*57:01 alleles from the genotyping chip data using SNP2HLA [22] with the Type 1 Diabetes Genetics Consortium (T1DGC) reference panel [21].

We then assessed the association between the SNP rs2395029 and case-control status separately in the four European cohorts using Fisher’s exact test. We also performed logistic regression analyses adjusted for the first two principal components (PCs) derived from PCA, which yielded similar results (not shown). The same association analyses were performed using imputed HLA-B*57:01 alleles, which produced nearly identical results (not shown). For this reason, both rs2395029 and HLA-B*57:01 are reported together in Table 1.

Association results were then combined across cohorts using both a global Fisher’s exact test and a fixed-effects meta-analysis, assuming a common effect size due to the comparable genetic backgrounds and study designs of the cohorts.

All statistical analyses were conducted in R (version 4.3.3). Fisher’s exact tests were performed using the base stats package, and the fixed-effects meta-analysis was carried out with the meta package (function metagen).

**Table 1:** Minor allele frequency of rs2395029-G/HLA-B*57:01* and association results across European cohorts.

Populations	Centenarians		Controls		Fisher	
	Number	MAF	Number	MAF	p value**	OR [95% CI]
**French**	1067	0,019	2438	0,029	0,01	0,65 [0.45;0.94]
**Germans**	692	0,024	6332	0,031	0,15	0,76 [0.51;1.09]
**Danish**	505	0,024	430	0,031	0,32	0,75 [0.41;1.36]
**Italians**	333	0,022	773	0,039	0,05	0,57 [0.29;1.09]
**Combined**	2597	0,022	9973	0,031	0,0002	0,69 [0.55;0.84]
**Meta-analysis**					0,0003	0.68 [0.55;0.84]

*rs2395029 was genotyped and HLA-B*57:01 was imputed and they gave quasi-identical results (see methods) **the p values were computed using Fisher exact test. Similar p values were obtained by logistic regression.

## RESULTS

We analyzed the allelic frequency of rs2395029-G in 4 independent cohorts of European centenarians coming from France [11], Germany [16], Denmark [20], and Italy [23]. Since the distribution of HLA class I alleles may vary across populations, we first analyzed the distribution of rs2395029-G separately within each cohort in comparison with geographically matched controls (Table 1). As presented in Table 1, the minor allele frequency (MAF) of rs2395029-G was consistently higher in control groups compared to centenarians, with odds ratios (OR) ranging from 0.57 to 0.76. Notably, this difference between cases and controls was statistically significant in the French and Italian populations. To evaluate the overall association of rs2395029-G/HLA-B*57:01 with extreme longevity, we performed a meta-analysis combining the 4 comparisons, yielding a significant OR of 0.68 (p=0.0003). Given the comparable MAFs between cases and controls across cohorts, we also pooled all case and control data (Table 1), yielding 2597 centenarians compared with 9973 controls, and found a very similar OR (=0.69) and p value (=0.0002).

## DISCUSSION

The present observation is important for several reasons. First, it is hard to conceive that the sole HLA class I presentation by the HLA-B*57:01 allele could be preventative of longevity. Indeed, HLA-B*57:01 is characterized by a relatively restricted peptide-binding repertoire yet exhibits high peptide-binding affinity [25], which may enhance presentation of viral peptides to the immune system, and has been associated with elite control of HIV-1 infection [1]. If this mechanism of high affinity presentation was relevant to human longevity, one would expect HLA-B*57:01 to be enriched among centenarians. However, our data demonstrate a statistically significant decrease in the frequency of HLA-B*57:01 within this age group. They rather support the hypothesis that dysfunction of the MICA/MICB axis associated with the HLA-B*57:01 haplotype provides a more plausible explanation for its decreased prevalence in long-lived individuals. The individuals carrying rs2395029-G/HLA-B*57:01 are approximately 6% of the general population in Europeans, whereas it is only 4% among European centenarians. This potentially indicates selection against carriers towards becoming a centenarian (one third lower chance), that could be due to the partially impaired NK cell defense against infectious diseases or cancers. Our results show slight differences between populations, with Germans and Danes exhibiting slightly different prevalences of HLA-B*57:01 compared to French and Italians (Table1). This variation is likely population-dependent and may be modulated by various factors such as ancestry, local pathogenic landscapes, or medical exposures, reflecting the dynamic co-evolution between host and environment. Therefore, it will be interesting to test the impact of this allele in other populations.

Second, this is the first genetic association demonstrating “in vivo” that robust innate immunity, precisely an efficient NK cell function, confers a longevity advantage. It would not be surprising if, in the general population, the lifespan of subjects carrying the HLA-B*57:01 would be globally shorter.

Third, this work emphasizes that for any HLA class I or class II gene association one should always consider the possibility of other molecular mechanisms beyond class I and class II antigen presentation, corresponding to the potential effects of other genetic variants in this LD rich, gene rich MHC region [5].
